# Behavioral Problems in Juvenile Idiopathic Arthritis: A Controlled Study to Examine the Risk of Psychopathology in a Chronic Pediatric Disorder

**DOI:** 10.1155/2016/5726236

**Published:** 2016-08-30

**Authors:** Amir Hossein Memari, Elham Chamanara, Vahid Ziaee, Ramin Kordi, Seyed-Reza Raeeskarami

**Affiliations:** ^1^Neuroscience Institute, Sports Medicine Research Center, Tehran University of Medical Sciences, Tehran, Iran; ^2^Children's Medical Center, Pediatrics Center of Excellence, Tehran, Iran; ^3^Pediatric Rheumatology Research Group, Rheumatology Research Center, Tehran University of Medical Sciences, Tehran, Iran; ^4^Department of Pediatrics, Tehran University of Medical Sciences, Tehran, Iran; ^5^Stanford University, Stanford, CA, USA; ^6^USG for Health, Treatment and Rehabilitation, Iranian Red Crescent Society, Tehran, Iran

## Abstract

Children with juvenile idiopathic arthritis (JIA) are prone to the problems that can delay their psychosocial development; however, the existing literature has not reached a consensus on the psychological problems related to JIA. A total of 51 children and adolescents with JIA and 75 healthy controls aged 6 to 18 years were examined using the Child Behavioral Checklist (CBCL). Our results represented that 70 percent of JIA group reached “borderline clinical” range or “clinical” range in internalizing problems, while this percentage in the control group was 18 percent. In addition, our results indicated that JIA group has gotten significantly higher scores (more than twofold) in externalizing behaviors compared to control group. Furthermore, children with JIA showed higher rate of anxiety/depression, withdrawal/depression, somatic complaints, rule breaking behaviors, and aggressive behaviors as well as thought and social problems compared to control group (*p* < 0.001). As a conclusion, children and adolescents with JIA compared to healthy controls may show higher rate of both internalizing and externalizing problems. Furthermore, our novel findings on externalizing, social, and thought problems in JIA warrant further investigation on affected children who may be at greater risk of future psychopathologies.

## 1. Introduction

Juvenile idiopathic arthritis (JIA) which consists of a heterogeneous group of chronic disorders (e.g., oligoarthritis, polyarthritis, and systemic arthritis) is an autoimmune, noninfectious inflammation of joints' synovial membrane and connective tissues that lasts more than 6 weeks [1, 2]. The disorder has been identified all over the world in nearly all races and ethnicities with an average prevalence rate of 1 to 2 per 1,000 children [3]. Some children may experience JIA symptoms for only a few months while others will suffer the symptoms and their consequences for the rest of their lives. In addition to pain and physical limitations, children with JIA may also experience high levels of stress during the course of their disease [4]. For example, they may find performing daily classroom activities challenging during periods of symptom exacerbation. Moreover, they may feel altered body image, anxiety around social acceptance, and fears about prognosis, treatment process, and their future. In addition, frequent visits to the doctor, limiting their leisure time, and restricting their activities may also add further stress on the affected child as well as his/her family. Adjustment problems have also been reported among children with JIA as well as their family. For example, children with active JIA may experience irritability, regression to more infantile patterns of behavior, loss of appetite, weight loss, and behavioral problems, particularly internalizing problems, additional to main physical symptoms. Furthermore, children with JIA experience more instances of sleep disturbances that may lead to behavioral and psychological consequences such as fatigue coupled with decrements in mood and performance [5, 6].

In fact, children with JIA are prone to the problems that can delay their psychosocial development; however, the existing literature has not reached a consensus on the psychological problems related to JIA. Rapoff et al. in a review study indicated that a few previous studies showed that psychosocial characteristics of children with JIA were comparable with healthy control populations [7]. This controversial data may show the methodological differences across previous studies with different psychological assessment tools [8]. Specifically, the majority of previous attempts consisted of small heterogeneous samples with different clinical status (e.g., varying state of the disease or heterogeneous joint involvement) that makes evaluation between studies difficult [9]. In order to compensate some of the limitations of previous studies, we conducted a case control study of youth with JIA but with no active arthritis. In fact, in order to remove the immediate effect of physical involvement and related disabilities, our samples consisted of children who were in remission phase.

On the other side, and to address the significance of this study, screening psychological or behavioral problems might help identifying children with JIA who are at risk of development of future psychopathologies. Several studies showed that mood disorders in childhood could predict continued risk for recurrences of depressive episodes and psychosocial morbidity into adulthood [10–12]. Furthermore, childhood behavior problems may adversely affect the academic performance of children with chronic disorders [13]. These associations hold for internalizing and externalizing problems in young children, as well as psychological distress and depression in older children [13]. Thus, a standard well known instrument, Child Behavioral Checklist (CBCL), was used to evaluate the maladaptive internalizing and externalizing behaviors. To our knowledge, there was no controlled study using CBCL in JIA population assessing internalizing and externalizing behavior problems. Furthermore, there is no data on children samples with JIA out of western societies. Thus, the aim of the current study is to investigate the association of JIA with behavioral problems in a sample of children from Iran.

## 2. Methods

### 2.1. Participants

The study consisted of a convenience sample of 51 children and adolescents with JIA (6–18 years old) who attended the rheumatology clinic at the Children's Medical Center, Tehran, Iran. During a period between April 2015 and November 2015, individuals with a diagnosis of JIA for at least 6 months but free of active arthritis at the time of the study were assigned to the study. Diagnosis of JIA for all participants was made by a rheumatologist. Patients were excluded if they had active arthritis, fever, serositis, splenomegaly or lymphadenopathy related to JIA, eye involvement, and abnormal C-reactive proteins or erythrocyte sedimentation rate. A group of 75 healthy children and adolescents (ages 6–18 years) were also enrolled into this study as the control group. We announced the study among a few schools in the hospital area and a researcher presented information about the project to selected classes. Considering the age and sex distribution of patient group, volunteers from healthy boys and girls were selected. Consequently, both patient and control groups were comparable in age (11.2 ± 3.5 in JIA versus 11.1 ± 3.2 in control group) and sex (Table 1). Finally, all participants came from Tehran (as the control group); and two group participants were matched on sex, age, and grade. Furthermore, all patients were selected from a pool of JIA patients attending rheumatology clinic with the same health care coverage. This study was approved by ethics committee of Tehran University of Medical Sciences. All parents and children participating in the study provided informed written consent. Parents and children completed assessments on background information and physical and psychological observations as well as a clinical assessment for children.

### 2.2. Disease Status

A rheumatologist assessed children and adolescents with JIA regarding active joints to identify disease status. Patients were also screened based on serological markers of inflammation including ESR and/or CRP. Participants in JIA group were controlled for eye involvement, serositis, and splenomegaly or lymphadenopathy. Disease duration in enrolled patients was in average 3.6 ± 2.3 years.

### 2.3. Child Behavioral Checklist

CBCL is a commonly used tool for identifying behavioral and emotional problems in children. This questionnaire belongs to the Achenbach System of Empirically Based Assessment developed by Thomas M. Achenbach. In the current study, we administered the school-age version of CBCL (CBCL/6–18) which is designed for children and adolescents aged 6–18 years. The parents/caregivers were asked to rate their children on 113 items based on response options, which included “*not true,*” “*somewhat or sometimes true,*” and “*very true or often true.*” The CBCL generates eight subscores and two composite internalizing and externalizing problem scores [14]. Problem behavior scores include withdrawal, somatic complaints, anxiety/depression, social problems, thought problems, attention problems (referring to characteristics of inattention, hyperactivity, and impulsivity), delinquent behavior, and aggressive behavior though scores may be summed into internalizing (e.g., anxious, withdrawn) and externalizing (e.g., aggressive, impulsive) behaviors. To interpret the CBCL data, using age and sex matched cut-offs, scores were classified into* normal* (scores that are below the 95th percentile),* borderline clinical range* (scores between the 95th percentile and 98th percentile), and* clinical range* (i.e., scores above the 98th percentile). According to previous published data, the CBCL validity and reliability were excellent, and extensive normative data are available for children ranging from 6 to 18 [15].

### 2.4. Statistics

Descriptive statistic was reported by mean and standard deviation of age and other dependent and independent variables. Problem behavior scores were presented as categorical data (Table 2), but to examine between-group differences and in order to compare the rate of internalizing and externalization problems between JIA and control group, we administered Mann–Whitney* U* test after normality test. Furthermore, to examine within-group differences of the rate of internalizing versus externalization problems (*t* scores), pair* t*-test analyses were conducted on JIA and control group separately. All statistical analyses were performed using SPSS version 17.0 and *p* value < 0.05 was considered statistically significant.

## 3. Results

Table 1 shows the demographic indices of both groups. As can be seen in Table 2, in regard to internalizing problems, our results represented that JIA group scored significantly higher compared to control group. Nearly 70 percent of JIA group reached borderline clinical range or clinical range in internalizing problems, while this percentage in the control group was 18 percent. In addition, our results indicated that JIA group has gotten significantly higher scores in externalizing behaviors as well. The percentage of individuals who reached the borderline clinical range and clinical range in externalizing problems was 48 and 21 percent in JIA and control group, respectively.

According to Mann–Whitney* U* analysis, subscales of anxiety/depression, withdrawal/depression, somatic complaints, rule breaking behaviors, and aggressive behaviors were scored in JIA significantly higher than control group (*p* < 0.001). The amount of thought and social problems was also greater in JIA group compared to control group (*p* < 0.001) while attention problems did not significantly differ between two groups (*p* > 0.05).

Moreover, in order to compare the internalizing problems with externalization problems in JIA or control group, we administered pair* t*-test analysis on* t* scores. The results showed that children with JIA scored 16.4 (8.0) in internalizing problems versus 20.4 (7.7) in externalizing problems (*t* = 2.49, *p* = 0.016). However, there was no difference between internalizing and externalizing problem scores in control group (*p* > 0.05).

## 4. Discussion

Our results indicated that, besides internalizing problems, children and adolescents with JIA also suffer from externalizing problems such as rule breaking and aggressive behaviors compared to healthy controls.

It should be noted that internalizing problems are characteristically associated with negative personality features such as neuroticism as well as a relatively overactive behavioral inhibition system (BIS) [16]. On the other hand, externalizing problems are often associated with novelty seeking traits and approach-related behavioral activation system (BAS) [16]. About the internalizing problems and JIA, Margetić et al. reported that children with JIA showed more depressive symptoms which in turn were associated with the level of pain they experienced [17]. Moreover, Feinstein et al. reported that pain and psychological inflexibility are associated with functional disability, maladjustment, anxiety, and lower level of quality of life in children with JIA [18]. To provide an explanation for this finding, some researchers described that one possibility is that the physical limitations and JIA-related symptoms may constrain normal “acting out” behaviors and actions seen in physically healthy children [19]. From the previous literature, there are also studies showing a normal status for both internalizing and externalizing behaviors in children with JIA [20]. For example, Noll et al. showed that although mothers reported more internalizing symptoms in their child with JIA, child self-reports and father reports showed no differences between children with JIA and controls [21]. LeBovidge et al. in a meta-analytic review also reported that youths with arthritis displayed increased risk for internalizing problems but not externalizing problems [19]. A possible explanation for the issue can be due to the duration from the onset of the disease in selected patients. Indeed, children are vulnerable during the early years following the diagnosis but can adapt in the longer term. Thus, our sample with shorter duration of JIA showed higher rate of internalizing or externalizing problems than studies examining individuals with longer duration of the disease [9].

Although there is almost no debate on the relationship between JIA and psychologic problems, our results provided new evidences supporting the idea that children with JIA compared to their healthy counterparts not only are at a greater risk of internalizing problems but also are more likely to suffer from externalizing problems. However, regarding the association between BIS and internalizing problems, one can argue that these children commonly internalize and inhibit their concerns and disease symptoms. Thus, as we showed, the risk of internalizing problems is still much higher than externalizing problems in JIA.

Current study also led to an interesting novel finding of higher rate of social and thought problems in JIA compared to the control group. This result corroborates the findings of a rare study by Reiter-Purtill et al. who in a controlled longitudinal study reported that children with severe or active JIA may be at risk for difficulties with social acceptance over time [22]. To provide an explanation, the disability-stress-coping model reflects that pediatric chronic disease (e.g., JIA) is an ongoing chronic strain for children and exposes them to negative life events while intrapersonal factors (e.g., problem solving ability), environmental factors (e.g., family resources), and stress processing factors (e.g., coping strategies) are their compensatory mechanisms against negative tensions. Thus, as Huygen et al. showed, children with JIA showed a high level of social adjustment when they were supported by highly cohesive family structures [23]. The “thought problems” scale that reflects symptoms that are usually found in mental disorders such as self-harm and strange thoughts and behaviors has received fewer/less attention than other subscales of Achenbach in JIA. As far as we know, we are the first showing thought problems in children and adolescents with JIA. It is worth mentioning that there are evidences representing that when thought problems are considered with rule breaking scale (part of externalization problems) or with the somatic complaints scale (part of internalizing problems), thought problems can be predictive for mood and psychotic problems. Thus, our findings warrant further studying long term risk of mood and psychotic problems in children with JIA if their behavioral problems receive no attention and remain untreated.

This study had some potential limitations that should be addressed. One of the limitations is that there were no other medical conditions (e.g., a chronic disease) as control group other than healthy group. This might help us to better rule out confounding factors related to physical symptoms and challenges shared in medical conditions.

## 5. Conclusion

The findings indicated that children and adolescents with JIA may not cope efficiently with the psychological consequences of their chronic condition and show higher rate of internalizing and externalizing problems. This maladjustment could interfere with their functions in social, behavioral, and even thought abilities. Thus, besides physical side of chronic disorders such as JIA, attention should be paid to the psychological side of their disease.

## Figures and Tables

**Table 1:** Demographic data of juvenile idiopathic arthritis and control groups.

	JIA (*n* = 51)	Control (*n* = 75)
Gender		
Male	23 (45%)	36 (48%)
Female	28 (55%)	39 (52%)

Children education		
Preschool	6 (11%)	9 (12%)
Elementary school	26 (50%)	39 (52%)
Middle school	11 (21%)	15 (20%)
High school	9 (17%)	12 (16%)

Parent's marital status		
Together	48 (94%)	72 (96%)
Separate	2 (4%)	1 (1%)
Widowed	1 (2%)	2 (2%)

**Table 2:** The rate of behavioral problems (ranging from normal to clinical range) measured by CBCL in both groups.

	Normal	Borderline clinical range	Clinical range
Anxiety/depression			
JIA^*∗*^	34 (66%)	10 (19%)	7 (13%)
Control	74 (98%)	1 (1%)	0 (0%)

Withdrawal/depression			
JIA	31 (60%)	6 (11%)	14 (27%)
Control	71 (94%)	3 (4%)	1 (1%)

Somatic complaints			
JIA	30 (58%)	15 (29%)	6 (11%)
Control	69 (92%)	6 (8%)	0 (0%)

Social problems			
JIA	33 (64%)	11 (21%)	7 (13%)
Control	68 (90%)	6 (8%)	1 (1%)

Attention problems			
JIA	37 (72%)	8 (16%)	6 (11%)
Control	58 (77%)	11 (14%)	6 (8%)

Thought problems			
JIA	35 (68%)	7 (13%)	9 (17%)
Control	69 (92%)	4 (5%)	2 (2%)

Rule breaking behavior			
JIA	39 (76%)	10 (19%)	2 (4%)
Control	67 (89%)	6 (8%)	2 (2%)

Aggressive behaviors			
JIA	38 (74%)	8 (15%)	5 (10%)
Control	68 (90%)	7 (9%)	0 (0%)

Externalization problems			
JIA	26 (50%)	10 (19%)	15 (29%)
Control	59 (78%)	9 (12%)	8 (9%)

Internalizing problems			
JIA	15 (29%)	9 (17%)	27 (53%)
Control	61 (81%)	11 (14%)	3 (4%)

^*∗*^Juvenile idiopathic arthritis.
